# Sex and gender differences in depressive symptoms in older workers: the role of working conditions

**DOI:** 10.1186/s12889-022-13416-1

**Published:** 2022-05-21

**Authors:** Sascha de Breij, Martijn Huisman, Cécile R. L. Boot, Dorly J. H. Deeg

**Affiliations:** 1 Department of Epidemiology and Data Science, Amsterdam UMC, Vrije Universiteit Amsterdam, Amsterdam Public Health Research Institute, De Boelelaan 1089A, 1081 Amsterdam, the Netherlands; 2 Department of Sociology, Vrije Universiteit Amsterdam, Amsterdam, the Netherlands; 3 Department of Public and Occupational Health, Amsterdam UMC, Vrije Universiteit, Amsterdam Public Health Research Institute, Amsterdam, the Netherlands

**Keywords:** Ageing, Inequalities, The Netherlands, Gender roles, Differential vulnerability hypothesis, Differential exposure hypothesis, Mental health, Job demands

## Abstract

**Background:**

Female older workers generally leave the work force earlier than men. Depressive symptoms are a risk factor of early work exit and are more common in women. To extend working lives, pathways leading to these sex inequalities need to be identified. The aim of this study was to investigate the association of sex and gender with depressive symptoms in older workers, and the role of working conditions in this association.

**Methods:**

We used data from the Longitudinal Aging Study Amsterdam (2012–2013/2015–2016, *n* = 313). Our outcome was depressive symptoms, measured by the Center for Epidemiologic Studies Depression Scale. We included biological sex, a gender index ranging from masculine to feminine (consisting of six items measuring gender roles: working hours, income, occupation segregation, education, informal caregiving, time spent on household chores), and working conditions (physical demands, psychosocial demands, cognitive demands, autonomy, task variation, social support) in our models. We examined the differential vulnerability hypothesis, i.e., sex/gender moderates the association between working conditions and depressive symptoms, and the differential exposure hypothesis, i.e., working conditions mediate the association between sex/gender and depressive symptoms.

**Results:**

Female sex and feminine gender were both associated with more depressive symptoms. The differential vulnerability hypothesis was not supported by our results. We did find that femininity was negatively associated with autonomy and task variation. In turn, these working conditions were associated with fewer depressive symptoms. Thus, autonomy and task variation partially mediated the association between gender and depressive symptoms, supporting the differential exposure hypothesis. Mediation effects for sex inequalities were not significant.

**Conclusions:**

Older female workers and older feminine workers have more depressive symptoms than their male/masculine counterparts. Autonomy and task variation appeared to be important in – partially – explaining gender differences in depressive symptoms rather than sex differences. By improving these conditions, gender inequality in mental health among older workers can be reduced, so that both genders have similar chances to reach the retirement age in good mental health.

Extending working lives is an important agenda of many countries. The labour force participation of (older) women continues to stay behind the participation rate of men [1]. The average duration of working life in the EU in 2019 was 38.3 years for men compared to 33.4 years for women [2]. Older female workers generally have more health problems compared to their male counterparts [3]. Sex differences are especially apparent in depression and depressive symptoms [4]. Depressive symptoms have been found to be associated with poor work outcomes, such as early exit from the labour market, disability, and loss of productivity [5–7]. Because sex differences are also present in working conditions [8], they may play a role in explaining sex inequalities in mental health.

It has been proposed that, while most research has focused on sex differences, a distinction should be made between sex and gender [9]. While sex is a biological construct, whereby an individual is defined as being male or female according to genetics, anatomy and physiology, gender is a social construct. Gender norms affect people’s behavior, perceptions of themselves and others and how they interact [10]. They can become manifest in gender roles. Gender roles “represent the behavioral norms applied to men and women in society, which influence individuals’ everyday actions, expectations, and experiences. Gender roles often categorise and define individuals within the family, the labour force, or the educational system” (10, p.3). The distinction between sex and gender is important because sex and gender may have a different effect on health and vulnerability to stressors [11–16].

Two hypotheses, that are not mutually exclusive, have been proposed as to how working conditions may play a role in sex and gender inequalities in health. The differential exposure hypothesis [17] posits that men and women are exposed to different working conditions. Women usually have jobs with more psychosocial demands, have less autonomy, and have less variation in their tasks compared to men [8, 18]. There is some evidence that gender (as measured by gender roles) differences in (perceived) working conditions are larger than sex differences [14]. Poor working conditions, such as low job control, high job demands, and low social support, have been found to be associated with poor mental health [19–23]. Therefore, they could be mediators of the relationship between sex and/or gender and health. Another hypothesis is the differential vulnerability hypothesis, which states that men and women react differently to the same working conditions and thus, their vulnerability to these risk factors differs (i.e. sex/gender is a moderator in the working conditions – health relationship) [17]. Some studies found that the health effects of social support at work were stronger for women than for men [24, 25], and that men benefit more from autonomy at work than women, in terms of health [25]. However, a meta-analysis on the effect of working conditions on depressive symptoms did not find support for sex differences in the magnitude of this association [21], which does not support the differential vulnerability hypothesis for this particular case.

Most studies on working conditions and sex inequalities in health so far have focused on the entire working population or on specific sectors. Research on older workers is scarce. To be able to extend working lives, a focus on older workers is necessary. Furthermore, most studies only focused on sex differences and neglected the role of gender. Although nowadays workers work up to higher ages with health problems compared to past decades, unhealthy workers still leave the labour market earlier than those in good health [26, 27]. Identifying determinants of poor mental health and clarifying underlying pathways leading to sex and gender inequalities in health is necessary to develop and implement interventions aimed at improving health of older workers and extending working lives for both sexes and genders. Therefore, the aim of the current study is to investigate 1) the association of sex and gender with depressive symptoms in older workers, and 2) the role of working conditions regarding these sex and gender inequalities, by testing the differential vulnerability hypothesis and the differential exposure hypothesis.

## Materials and methods

### Sample and design

We used data from the Longitudinal Aging Study Amsterdam (LASA). LASA is an ongoing, prospective cohort study, based on a representative sample of the older population in the Netherlands. LASA focuses on the determinants, trajectories and consequences of changes in physical, cognitive, emotional, and social functioning in older adults aged 55 years or older. Measurements are conducted approximately every three years and include a main face-to-face computer assisted interview, a face-to-face computer assisted medical interview in which clinical measurements are performed and additional questions are asked, and a self-administered questionnaire. The study received approval by the medical ethics committee of the VU University medical center. Signed informed consent was obtained from all study participants. Sampling, response and procedures are described in detail elsewhere [28].

For the current study, we adopted a lagged-effect design, because we expected that with ageing, older workers would increasingly be affected by their gender role (our main determinant) and working conditions (our moderator/mediator), and that this would result in higher depressive symptoms scores in the course of time. Thus, we assumed a temporal precedence of gender roles and working conditions, as opposed to an immediate effect on depressive symptoms. Accordingly, data from 2012–2013 (T1) and 2015–2016 (T2) were used. At T1, 1023 respondents participated in the LASA study. We excluded those who did not have a paid job at T1 (*n* = 395), those who did not participate at T2 (*n* = 93), and those who did not have a paid job at T2 (*n* = 222). We ended up with a sample of 313 older workers.

### Measures

#### Outcome

Our outcome measure was depressive symptoms, measured using the Center for Epidemiologic Studies Depression Scale (CES-D) [29]. The CES-D is a 20-item self-report scale ranging from 0 to 60, with higher scores reflecting more depressive symptoms. The outcome was measured at T2 (2015/2016).

#### Independent variables

All independent variables were measured at T1 (2012/2013).

##### Sex

We included *biological sex,* derived from the population registers, as an independent variable.

##### Gender

We constructed a gender index, based on the work of Smith and Koehoorn [9] on gender roles in the labour market. Smith and Koehoorn included four gender items in their index: responsibility for caring for children, occupation segregation, number of working hours, and level of education. Because in our sample of older workers responsibility for caring for children was not applicable, we chose to include a measure of informal caregiving. Providing informal care is much more common among women compared to men and is seen as a more feminine role [30, 31]. As suggested by Smith and Koehoorn, we also included a measure of household responsibilities. Furthermore, Smith and Koehoorn suggested to include a measure for primary earner status. Unfortunately this information was not available in our data. We therefore chose to include income in our index. While Smith and Koehoorn use relative measures (relative to the partner) for educational level and number of working hours, we use absolute measures for these items, because we consider absolute measures to reflect broader societal gender roles rather than gender roles within the household.

The gender index consisted of the sum score of six items: number of working hours, income, occupation segregation, level of education, informal caregiving, and time spent on household chores. For each gender item, a higher score represents more femininity and a lower score represents more masculinity.

Respondents were asked about their *number of working hours* per week. Responses were categorised into quartiles and recoded so that a higher score (i.e. a lower number of working hours) represents more femininity.

To assess the *income* of the household, respondents were asked what their monthly household income was, choosing from 24 categories, with the lowest category being €454-€567 and the highest category €5446 or more. To ensure comparability of income between persons with and without a partner in the household, income was multiplied by 0.7 for respondents with a partner in the household. The factor 0.7 is the inverse of the squareroot of 2, i.e., the number of household members. This correction makes the incomes of all respondents equivalent to one-person household incomes [32]. Income was categorised into quartiles and recoded so that a higher score (i.e. a lower income) represents more femininity.

*Occupation segregation* was measured by the percentage of female workers in the sector. Using data from Statistics Netherlands, we assigned each sector to one of four categories in accordance with Smith and Koehoorn [9]: (0) ≤ 25% female workers, (1) 26–50% female workers, (2) 51–75% female workers, and (3) ≥ 76% female workers.

Respondents were asked about their highest completed level of education. We used the International Standard Classification of Education 2011 [33] to categorise *educational level* into three groups: (0) low (up to lower secondary education, ISCED 0–2), (1) intermediate (upper secondary education or post-secondary non-tertiary education, ISCED 3–4), and (2) high (short cycle tertiary and higher, ISCED 5–6). Again, scores were recoded so that a higher score (i.e. lower educational level) reflects more femininity.

Respondents were asked if they recently provided help with household chores to somebody outside the own household, and whether the respondent provided help with personal care to somebody inside or outside the own household. If so, questions were asked about the intensity (hours) of care. *Informal caregiving* was categorised into (0) not giving informal care, (1) giving < 8 h of informal care per week, and (2) giving ≥ 8 h of informal care per week.

Respondents were also asked about the time spent on light and heavy *household chores*. Time in minutes per day, averaged across the past 14 days, was categorised into quartiles.

The gender index ranged from 0–22 and was dichotomised at the median into masculine (scores 0–7) and feminine (scores 8–22) to enable comparison of its association with depressive symptoms with the association of biological sex with depressive symptoms.

##### Working conditions

We used a written questionnaire to obtain data on working conditions [34]. Respondents could answer (1) never, (2) sometimes, (3) often, or (4) always to all questions on working conditions.

To measure *physical demands* five items were used: ‘use of force’, ‘using tools that cause vibration or shaking’, ‘working in an uncomfortable position ‘, ‘standing for a long time’, and ‘kneeling down or squatting’. *Psychological demands* consisted of two items: ‘working very fast’, and ‘having to do a lot of work’. For *cognitive demands*, six items were used: ‘think of solutions’, ‘learn new things’, ‘requires creativity’, ‘requires thinking intensively’, ‘requires focus’, and ‘ requires attention’. *Autonomy* was measured with three items: ‘control over how to do the job’, ‘control over sequence of tasks’, and ‘control over when to take time off’. For *variation in tasks* one item was used: ‘having variation in tasks’. And for *social support* four items were included: ‘help and support of colleagues’, ‘colleagues willing to listen to work related problems’, ‘help and support of supervisor’, and ‘supervisor willing to listen to work related problems’.

Sum scores were made for each type of working conditions and scores were dichotomised using the median due to non-linearity.

##### Control variables

Age was derived from the population registers.

### Statistical analysis

**Fig. 1:**
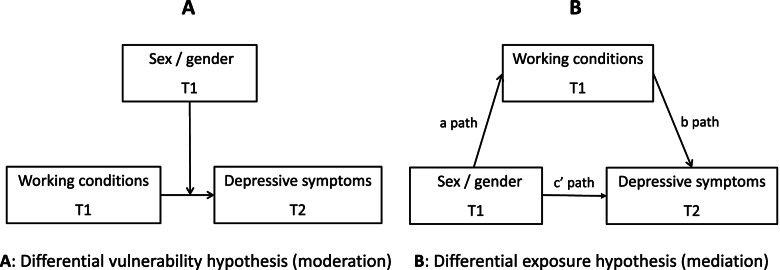
Visual representation of the moderation and mediation models

Multiple imputation (MICE) was used to deal with missing values, which were assumed to be missing at random. All independent, control and the outcome variables were included in the imputation process and the number of imputations was set to 30, based on the percentage of missing values (28%) [35]. To assess to what extent the separate gender items as well as the gender index are associated with sex, we conducted logistic regression analyses [36]. We used Structural Equation Modeling (SEM) to estimate the associations visualised in Fig. 1. All analyses were adjusted for age. Separate models were examined for sex and gender. We used tobit regression analyses to estimate the associations of sex/gender and the working conditions with depressive symptoms, because the depressive symptoms scale is skewed to the right due to the detection limit at the lower end of the scale. Tobit models account for this left-censoring by assuming a normal distribution that is cut off (censored) at zero.

#### Differential vulnerability hypothesis

To test whether gender/sex is a moderator in the association between working conditions and depressive symptoms, we built models with an interaction between sex/gender and the working conditions (Fig. 1A). In case of a statistically significant interaction, the association between the working conditions and depressive symptoms varies across sexes/genders.

#### Differential exposure hypothesis

To investigate whether working conditions explain the association between sex/gender and depressive symptoms, we built single mediator analyses (Fig. 1B). To estimate the c paths (total effect of sex/gender on depressive symptoms) and the b paths (effect of the mediators on depressive symptoms, while controlling for sex/gender), we used tobit regression analyses, and for the a paths (the effect of sex/gender on the mediators), we conducted logistic regression analyses. We used causal mediation analyses to estimate the indirect effects [37]. We used bootstrapping techniques (500 repetitions) to calculate the 95% confidence intervals around the indirect effects. All analyses were carried out in Stata version 14.

## Results

Table 1 shows the characteristics of the sample. 18.1% of men and 70.6% of women were categorised as feminine on the gender index.

**Table 1:** Characteristics of the sample

	**Male (*n* = 177)** **%**	**Female (*n* = 136)** **%**	**Masculine (*n* = 185)** **%**	**Feminine (*n* = 128)** **%**	**Total (*n* = 313)** **%**
Age, M(SD)	59.0 (2.4)	58.5 (2.4)	58.9 (2.4)	58.6 (2.4)	58.0 (2.4)
Female	0.0	100.0	21.6	75.0	43.5
Feminine	18.1	70.6	0.0	100.0	40.9
Income (quartiles)					
*Q1 (€510–1900)*	20.7	32.8	11.9	46.0	26.0
*Q2 (€1900–2399)*	22.5	26.7	18.8	32.3	24.3
*Q3 (€2400–2999)*	28.4	25.2	33.5	17.7	27.0
*Q4 (*≥ *€3000)*	28.4	15.3	35.8	4.0	22.7
Educational level					
*Low*	29.9	29.4	19.5	44.5	29.7
*Intermediate*	24.3	30.9	22.2	34.4	27.2
*High*	45.8	39.7	58.4	21.1	43.1
Informal caregiving					
*None*	83.2	62.3	87.1	55.6	74.1
< *8 h per week*	13.4	26.3	12.3	28.7	19.0
≥ *8 h per week*	3.4	11.4	0.6	15.7	6.8
Time spent on household chores (quartiles)					
*Q1 (0–24 min per day)*	42.4	5.9	40.5	6.3	26.5
*Q2 (24–60 min per day)*	29.9	16.9	29.2	17.2	24.3
*Q3 (60–120 min per day)*	15.8	35.3	21.1	28.9	24.3
*Q4 (*≥ *120 min per day)*	11.9	41.9	9.2	47.7	24.9
Working hours (quartiles)					
*Q1 (1–20 h per week)*	11.4	43.4	8.7	49.2	25.3
*Q2 (20–35 h per week)*	18.2	34.6	22.0	28.9	25.3
*Q3 (35–41 h per week)*	52.3	17.6	48.9	20.3	37.2
*Q4 (*≥ *41 h per week)*	1.2	4.4	19.6	1.6	12.2
Occupation segregation (% female workers)					
≤ *25%*	41.2	8.1	41.6	5.5	26.8
*26–50%*	27.1	11.0	25.9	11.7	20.1
*51–75%*	27.7	65.4	29.7	64.8	44.1
≥ *76%*	4.0	15.4	2.7	18.0	8.9
High physical demands	42.9	50.0	36.2	60.2	46.0
High psychosocial demands	44.1	44.1	44.3	43.8	44.1
High cognitive demands	38.4	33.1	45.4	22.7	36.1
High autonomy	45.8	36.0	47.6	32.8	41.5
High variation in tasks	40.3	34.1	42.5	30.6	37.5
High social support	38.4	36.0	38.9	35.2	37.4
Depressive symptoms, M(SD)	5.1 (5.4)	6.5 (5.8)	5.0 (5.4)	6.6 (5.7)	5.7 (5.6)

### Associations between sex and gender

The logistic regression analyses showed that the separate gender items were all associated with biological sex, except for educational level (Table 2). A higher income and a higher number of working hours was associated with male sex whereas providing informal care, spending more time on household chores, and having an occupation with a majority of female workers was associated with female sex. The gender index constructed from these items also showed a statistically significant association with biological sex (OR = 10.88, 95%CI = 6.39;18.51).

**Table 2:** Association between the separate gender items and sex

	**OR (95%CI)**
Income (quartiles)	
*Q1 (€510–1900)*	R
*Q2 (€1900–2399)*	0.75 (0.40;1.42)
*Q3 (€2400–2999)*	0.56 (0.30;1.05)
*Q4 (*≥ *€3000)*	0.34 (0.17;0.67)**
Educational level	
*Low*	R
*Intermediate*	1.29 (0.72;2.34)
*High*	0.88 (0.52;1.51)
Informal care	
*None*	R
< *8 h per week*	2.62 (1.39;4.95)**
≥ *8 h per week*	4.54 (1.55;13.26)**
Time spent on household chores (quartiles)	
*Q1 (0–24 min per day)*	R
*Q2 (24–60 min per day)*	4.07 (1.69;9.79)**
*Q3 (60–120 min per day)*	16.07 (6.77;38.18)**
*Q4 (*≥ *120 min per day)*	25.45 (10.51;61.60)**
Working hours (quartiles)	
*Q1 (1–20 h per week)*	R
*Q2 (20–35 h per week)*	0.50 (0.25;0.90)*
*Q3 (35–41 h per week)*	0.09 (0.04;0.17)**
*Q4 (*≥ *41 h per week)*	0.06 (0.02;0.17)**
Occupation segregation (% female workers)	
≤ *25%*	R
*26–50%*	2.07 (0.88;4.90)
*51–75%*	12.05 (5.85;24.85)**
≥ *76%*	19.91 (6.87;57.73)**

^*^p < 0.05

^**^p < 0.01

### Associations between sex/gender and depressive symptoms

Female sex (B = 1.70, 95%CI = 0.29;3.11) and femininity (B = 1.85, 95%CI = 0.43;3.26) were both statistically significantly associated with more depressive symptoms.

#### Differential vulnerability hypothesis

We then added interaction terms to examine whether sex and gender were effect modifiers in the associations between working conditions and depressive symptoms. None of the interaction terms were statistically significant. We therefore concluded that the differential vulnerability hypothesis was not confirmed by our data (Table 3).

**Table 3:** Interaction between working conditions and sex/gender with regard to depressive symptoms

	**Sex**			**Gender**		
	Main effect working condition B (95%CI)^a^	Main effect sex B (95%CI)^a^	Interaction working condition*sex	Main effect working condition B (95%CI)^a^	Main effect gender B (95%CI)^a^	Interaction working condition*gender
Physical demands	0.71 (-1.18;2.60)	2.59 (0.66;4.52)**	-1.89 (-4.72;0.94)	0.17 (-1.72;2.07)	2.73 (0.67;4.80)*	-1.54 (-4.45;1.37)
Psychosocial demands	0.57 (-1.30;2.44)	2.75 (0.88;4.62)**	-2.41 (-5.23;0.41)	0.17 (-1.66;2.01)	2.53 (0.64;4.42)**	-1.57 (-4.42;1.27)
Cognitive demands	0.22 (-1.71;2.14)	1.85 (0.09;3.61)*	-0.43 (-3.37;2.52)	1.16 (-0.66;2.99)	2.62 (0.88;4.37)**	-2.23 (-5.38;0.92)
Autonomy	-1.68 (-3.54;0.19)	1.51 (-0.30;3.32)	0.06 (-2.81;2.93)	-1.87 (-3.68;-0.05)*	1.33 (-0.48;3.14)	0.76 (-2.17;3.69)
Variation in tasks	-2.74 (-4.67;-0.81)**	1.10 (-0.67;2.86)	1.13 (-1.78;4.05)	-2.08 (-3.94;-0.22)*	1.71 (-0.04;3.47)	-0.11 (-3.08;2.85)
Social support	-0.58 (-2.48;1.33)	2.31 (0.55;4.07)*	-1.78 (-4.67;1.11)	-1.27 (-3.14;0.60)	1.84 (0.07;3.62)*	-0.15 (-3.07;2.78)

men/masculine and low exposure to working conditions are the reference groups

^a^B adjusted for age

^*^p < 0.05

^**^p < 0.01

#### Differential exposure hypothesis

Next, we built single mediator models to investigate the mediating role of working conditions in the association of sex and gender with depressive symptoms (Table 4).

**Table 4:** Single-mediator models of working conditions in the association between sex/gender and depressive symptoms

	**Sex**				**Gender**			
**Mediator**	a path B (95%CI)^a^	b path B (95%CI)^a^	c’ path B (95%CI)^a^	Indirect effect B (95%CI)^a^	a path B (95%CI)^a^	b path B (95%CI)^a^	c’ path B (95%CI)^a^	Indirect effect B (95%CI)^a^
Physical demands	0.30 (-0.15;0.75)	-0.13 (-1.53;1.27)	1.71 (0.30;3.12)*	-0.04 (-0.72;0.57)	1.00 (0.53;1.46)**	-0.48 (-1.91;0.96)	1.96 (0.51;3.42)**	-0.48 (-2.08;0.94)
Psychosocial demands	-0.02 (-0.48;0.43)	-0.49 (-1.90;0.91)	1.70 (0.29;3.11)*	0.01 (-0.54;0.37)	-0.04 (-0.50;0.41)	-0.48 (-1.88;0.92)	1.84 (0.43;3.25)*	0.02 (-0.36;0.57)
Cognitive demands	-0.21 (-0.68;0.26)	0.04 (-1.41;1.49)	1.70 (0.29;3.11)*	-0.01 (-0.61;0.45)	-1.03 (-1.54;-0.53)**	0.42 (-1.07;1.90)	1.94 (0.49;3.39)**	-0.43 (-2.02;1.16)
Autonomy	-0.41 (-0.87;0.05)	-1.65 (-3.06;-0.24)*	1.53 (0.13;2.94)*	0.68 (-0.07;1.79)	-0.63 (-1.09;-1.56)**	-1.57 (-2.99;-0.16)*	1.62 (0.20;3.04)*	0.98 (0.09;2.32)*
Variation in tasks	-0.24 (-0.72;0.25)	-2.24 (-3.68;-0.80)**	1.51 (0.11;2.92)*	0.53 (-0.52;2.07)	-0.50 (-1.00;-0.01)*	-2.13 (-3.57;-0.68)**	1.67 (0.26;3.09)*	1.07 (0.03;2.64)*
Social support	-0.14 (-0.61;0.33)	-1.35 (-2.79;0.09)	1.65 (0.25;3.05)*	0.19 (-0.58;1.07)	-0.19 (-0.66;0.28)	-1.33 (-2.77;0.10)	1.79 (0.38;3.20)*	0.25 (-0.38;1.34)

^a^B adjusted for age

^*^p < 0.05

^**^p < 0.01

We found that autonomy and variation in tasks partially mediated the association between gender and depressive symptoms. In the a path, femininity was positively associated with physical demands (B = 1.00, 95%CI = 0.53;1.46), and negatively associated with cognitive demands (B = -1.03, 95%CI = -1.54;-0.53), autonomy (B = -0.63, 95%CI = -1.09;-0.56), and variation in tasks B = -0.50, 95%CI = -1.00;-0.01). In turn, in the b path, high levels of autonomy (B = -1.57, 95%CI = -2.99;-0.16) and variation in tasks (B = -2.13, 95%CI = -3.57;-0.68) were associated with fewer depressive symptoms, but physical and cognitive demands were not. The indirect effects of autonomy and task variation were substantial (B = 0.98, 95%CI = 0.09;2.32 and B = 1.07, 95%CI = 0.03;2.64, respectively).

For the association between sex and depressive symptoms we found no mediators. No significant associations were observed in the a path. In the b path, autonomy (B = -11.65, 95%CI = -3.06;-0.24) and task variation (b = -2.24, 95%CI = -3.68;-0.80) were associated with depressive symptoms. None of the indirect effects were significant.

The mediation effects for the association between gender and depressive symptoms were only partial: even after controlling for these mediators, the direct effect (c’ path) of sex/gender on depressive symptoms remained substantial (and statistically significant). Therefore, these results partially support the differential exposure hypothesis.

## Discussion

The aim of this study was to investigate the association of sex and gender with depressive symptoms in older workers, and to examine the role of working conditions regarding these sex and gender inequalities, by testing two hypotheses: the differential vulnerability hypothesis and the differential exposure hypothesis. Our results first show that there was a strong association between biological sex and gender as measured by an index including stereotypical gender roles, i.e. number of working hours, income, occupation segregation, level of education, informal caregiving, and time spent on household chores. This association indicates that these gender roles are still very much embedded in society. If one would assume equal pay between men and women, equal household and care responsibilities, and equal employment conditions, 50% of men and women would be characterised as masculine on this index and 50% as feminine. However, in our sample, 18.1% of men and 70.6% of women were categorised as feminine. These findings support the persistence of gender stereotypes, despite some progress toward egalitarianism in the last decades, as described by Haines et al. [38].

Both biological sex and gender were associated with depressive symptoms, with women and those categorised as feminine being disadvantaged. Although sex differences in depressive symptoms have been well established [4], gender differences have been largely neglected. Our results show that, in older workers, the gender inequalities may be even larger than the sex inequalities (although in our sample their confidence intervals overlap).

We investigated two hypotheses that could give insight into the role of working conditions in sex/gender inequalities in depressive symptoms: the differential vulnerability hypothesis and the differential exposure hypothesis. In line with the meta-analysis by Theorell et al. [21], we did not find support for the differential vulnerability hypothesis, as indicated by the absence of a statistically significant interaction effect between sex/gender and working conditions in our data. Thus, we did not find support that sex/gender differences in depressive symptoms can be attributed to sex/gender differences in the effect of working conditions on depressive symptoms. We did, however, find partial support for the differential exposure hypothesis, in line with previous studies on several mental health outcomes [18–20]. Workers categorised as feminine experienced less autonomy and less variation in tasks than workers categorised as masculine, which in turn is associated with more depressive symptoms.

Gender differences in perceived working conditions were generally larger than sex differences, highlighting the importance of including gender and sex as separate constructs in studies on working conditions. This finding is in line with findings from a recent study by Kerr et al. [14]. In our study, gender was operationalised as gender roles, including income and educational level. Evidence shows that workers who earn lower wages and have lower educational levels, have higher job demands and less psychosocial resources at work, even after controlling for sex [39]. It may be that the sex differences in working conditions as found in previous studies may actually be (partly) due to gender roles. In such studies, biological sex serves as a proxy for gender. Our study, in contrast, shows the benefit of capturing gender roles directly.

There is an obvious overlap between labour-market gender and socio-economic position, as women of the generation studied achieved lower levels of education, and work in lower-level jobs with lower wages even compared to their male peers. This raises the question if the difference in depressive symptoms that we find should be interpreted as based on gender or rather on socio-economic position. Vice versa, reports on an association between socio-economic position and depressive symptoms among older workers might actually be rooted in labour-market gender status. The several indicators of our gender index mutually reinforce one another, and it is the composite score on the six indicators that together represents gender roles relevant to the labour market. Moreover, the social position of both women and men is at stake, and the implications regarding improvement of working conditions to extend working lives run in the same vein [40].

Especially in these times, with gender inequality widening due to the global COVID-19 pandemic [41] and new trends in remote and hybrid work, it is important for employers to recognise the effects of psychosocial resources at work. By improving autonomy and variation in tasks, sex and gender inequalities in depressive symptoms may be reduced.

This study has several limitations. First, we constructed a gender index by taking the index proposed by Smith and Koehoorn [9] as a starting point. We adapted their gender index based on their recommendations, availability of our data, and to reflect broader societal relevance. The validity of our gender index should be tested in other datasets and other national contexts. Gender differences in educational attainment and income vary across countries [9]. Regarding educational attainment, the sex difference has decreased in the past decades. In future studies, it may be an option to exclude education from the labour market gender index [42]. Furthermore, the Netherlands is known for its relatively large proportion of part-time female workers [43]. As ony a small minortity of men work part-time, this may widen the gender gap among older workers in terms of earnings, women’s slower progression into management roles, and an unequal division of unpaid work at home [44]. Also, gender differences in mental health vary across countries [45, 46], and country-specific policies and institutions may affect the health of women and men in different ways [47]. For these reasons, similar studies in other national contexts are recommended. Also, by summing the gender items to an index, we made assumptions about the relative contribution of each of the components. The weighting of items is also subject to further research, as is the use of the gender index as a continuum rather than as a dichotomy.

Second, besides gender roles, examining gender identity may give added insight into gender differences [10]. Unfortunately, no data on gender identity were available in our dataset.

Third, reversed causation may be an issue, i.e. depressive symptoms may affect (perceived) working conditions instead of the other way around. However, depressive symptoms were measured at T2 while the working conditions were measured at T1. Also, previous research on working conditions and mental health shows that, in this context, reversed causation is not likely [48, 49].

Fourth, in our study we focused on (modifiable) working conditions as possible mediators. While some of these working conditions indeed mediated the association between gender and depressive symptoms, a large part of the inequalities remained unexplained. In a larger dataset, multiple mediator models could be built to estimate the total mediating effect of multiple working conditions. Other work characteristics such as type of employment and emotional demands may also play a mediating role. In addition, the literature on sex differences in depression in the general population suggests a range of other possible mediators, e.g., negative life events such as widowhood, chronic illness, coping styles, emotional support [50, 51].

Fifth, our results may be influenced by the healthy worker effect, i.e. healthy individuals are more likely than their unhealthy peers to (still) be active in the labour market [52]. Since women leave the work force earlier and more often than men due to mental health issues [53], our specific sample may show weaker associations than a younger sample of workers would.

Sixth, due to collinearity we could not build models which included both sex and gender to disentangle their effects. Therefore, the effect of gender may be partly due to biological sex and vice versa.

Last, but not least, our sample is relatively small, which may limit generalizability even though it is population-based. In order to support generalizability to the general population of older workers, we compared the work participation of our cohort with the work participation for the general population as provided by Statistics Netherlands (SN). This comparison could be made for the subsample with birth years 1950–1954 at the age of 60 years, which is the average age of our cohort at baseline. We found very similar, if not slightly higher, percentages: Men (SN) = 71.7%, Men (LASA) = 73.8%; Women (SN) = 48.9%, Women (LASA) = 52.6%. Regardless, our study may be underpowered. On the positive side, this implies that the associations that we did find are certainly meaningful. In particular, the working conditions that proved to be mediators, i.e., autonomy and task variation, correspond to working conditions that were found to be mediators in the association between education and health across several countries in an earlier study [40]. Also, these working conditions emerged as significant factors to continue work participation in a qualitative study among chronically ill older workers [54].

## Conclusion

In conclusion, our results suggest that sex and gender differences in depressive symptoms are not due to a differential vulnerability to working conditions. Gender differences, however, can be partially attributed to a differential exposure to autonomy and task variation. Implementing workplace interventions targeted at improving these conditions may lead to a reduction of gender inequality in depressive symptoms among older workers, so that both genders have similar chances to reach the retirement age in good mental health.

## Data Availability

The data underlying the results presented in this study are available from the Longitudinal Aging Study Amsterdam (LASA). Data may be requested for research or replication purposes. Please contact the scientific director: ma.huisman@amsterdamumc.nl. More information on data requests can be found on the LASA website: www.lasa-vu.nl.

## Abbreviations

LASA: Longitudinal Aging Study Amsterdam
CES-D: Center for Epidemiologic Studies Depression Scale
ISCED: International Standard Classification of Education
